# Reply to ‘Comment on “The myth of pulmonary metastasectomy’”

**DOI:** 10.1038/s41416-020-01062-6

**Published:** 2020-09-22

**Authors:** Fergus Macbeth, Dame Lesley Fallowfield

**Affiliations:** 1 Centre for Trial Research, Cardiff University, Cardiff, UK; 2 Sussex Health Outcomes Research & Education (SHORE-C), Brighton & Sussex Medical School, University of Sussex, Falmer, BN1 9RR UK

**Keywords:** Surgical oncology, Colorectal cancer

We thank Zellweger and Gonzalez for their comments on our article about pulmonary metastasectomy.^1^ We agree with much of what they say about the need for multidisciplinary management and the importance of ruling out other diagnoses such as primary lung cancer, but we need to correct several misunderstandings. The results of all 93 randomised patients in PulMiCC have now been published in an updated report,^2^ which confirms the lack of a significant survival difference (hazard ratio (HR) 0.93 (95% confidence interval (CI): 0.56,1.56)) and median survivals of 3.5 and 3.8 years for intervention and control patients, respectively. Although the numbers randomised were small, the trial has sufficient power to make it highly improbable that the 5-year survival rate in unoperated patients is <5%, as is so widely believed.

Many reports and reviews of pulmonary metastasectomy cite that low, <5%, estimate without any valid evidential support; worse still, many quote the comparatively poor survival of patient groups, which are not comparable because they do not have same prognostic features as those selected for metastasectomy. An example is the retrospective study by Kim et al.,^3^ in which the comparator patients were significantly different in several important prognostic factors. PulMiCC is the only randomised controlled trial (RCT) with a truly comparable control population and has clearly shown that survival without metastasectomy is not as low as is so widely suggested. It also raises a serious question as to whether or not the oft reported good survival of those having pulmonary metastasectomy might be due to selection alone.

In their landmark meta-analysis of prognostic factors following pulmonary metastasectomy Gonzalez et al.^4^ included data from 25 reports. Of the 2600 for whom the data are available, 63% had a solitary metastasis, a strongly favourable prognostic factor, and 5-year survival was 41% when the data for the total 2925 patients (in Table 1 of Gonzalez et al.) are aggregated. PulMiCC included 32 (34%) patients with solitary metastases. The overall estimated 4-year survivals were 44% (operated) and 47% (controls)—survival figures in both arms are comparable with those estimated from Gonzalez et al.,^4^ despite PulMiCC having a lower proportion with solitary metastasis.

The control patients were not ‘untreated’ but managed by their local teams and 49% went on to have chemotherapy, as did 41% in the metastasectomy arm. Consequently, the controls did not, as implied, have any special treatment to account for their survival compared with non-trial patients with metastatic colorectal cancer.

Zellweger and Gonzalez do concede that ‘the survival benefit for surgical patients is probably modest’ but are ‘…certain that some patients will really benefit from curative surgical management’ because ‘we observe daily that single or multiple pulmonary metastasectomies may result in long-term survival or even cure’. Unfortunately, anecdote and strong belief are not substitutes for high-quality evidence and may, as has been seen often in the past, lead to misguided or unnecessary treatment.^5^

They describe the difficulty of carrying out an RCT when only one option is ‘potentially curative’ and this illustrates a very real problem—namely, the lack of equipoise. If the clinicians involved with explaining trial options to patients before trial enrolment are certain that only one option offers a chance of cure and therefore present the study to them in that way, then recruitment will of course be difficult. A study of a sample of those registered but not randomised in PulMiCC shows that if the patients themselves chose which treatment to have, about half opted not to have surgery, whereas if the surgical team decided then almost all were operated on.^6^ This contrasts with the assertion by Zellweger et al. that ‘many patients chose to undergo surgery’.

We believe that the results of PulMiCC provide sufficient evidence to generate doubt about the benefit of pulmonary metastasectomy. Thus, a further large definitive RCT is both ethical and essential. Unless the clinicians involved in such a trial can adjust their mindsets, exhibit genuine equipoise around the question and be more honest with patients about the lack of clear evidence for the benefit of metastasectomy, then the project is doomed to fail. Zellweger and Gonzalez think that giving ‘desperate’ patients hope is a sufficient justification for surgery. But giving patients hope with an uncertain promise of cure seems inappropriate. Evidence suggests that however well intentioned, giving patients misleading information may in fact be harmful.^7^

## Data Availability

Not applicable.
